# A monoclonal antibody raised against a thermo-stabilised β_1_-adrenoceptor interacts with extracellular loop 2 and acts as a negative allosteric modulator of a sub-set of β_1_-adrenoceptors expressed in stable cell lines

**DOI:** 10.1016/j.bcp.2017.10.015

**Published:** 2018-01

**Authors:** Mark Soave, Gabriella Cseke, Catherine J. Hutchings, Alastair J.H. Brown, Jeanette Woolard, Stephen J. Hill

**Affiliations:** a Division of Physiology, Pharmacology and Neuroscience, School of Life Sciences, University of Nottingham, Nottingham NG7 2UH, UK; b Centre of Membrane Proteins and Receptors (COMPARE), University of Birmingham and University of Nottingham, Midlands, UK; c Heptares Therapeutics Ltd., Bio Park, Welwyn Garden City AL7 3AX, UK

**Keywords:** Alprenolol (Pubchem CID: 2119), CGP 12177 (Pubchem CID: 2687), CGP 20712A (Pubchem CID: 2685), Cimaterol (Pubchem CID: 2755), Furimazine (Pubchem CID: 219083), Hoechst 33342 (Pubchem CID: 1464), IBMX (Pubchem CID: 3758), ICI 118551 (Pubchem CID: 5484725), Isoprenaline (Pubchem CID: 5807), Propranolol (Pubchem CID: 4946), ATCM, allosteric ternary complex model, CHO, Chinese hamster ovary, CRE, cAMP response element, ECL, extracellular loop, HEK, human embryonic kidney, mAb, monoclonal antibody, SPAP, secreted placental alkaline phosphatase, StaR, stabilised receptor, GPCR, Allosterism, Monoclonal antibody, Extracellular loop 2

## Abstract

Recent interest has focused on antibodies that can discriminate between different receptor conformations. Here we have characterised the effect of a monoclonal antibody (mAb3), raised against a purified thermo-stabilised turkey β_1_-adrenoceptor (β_1_AR-m23 StaR), on β_1_-ARs expressed in CHO-K1 or HEK 293 cells. Immunohistochemical and radioligand-binding studies demonstrated that mAb3 was able to bind to ECL2 of the tβ_1_-AR, but not its human homologue. Specific binding of mAb3 to tβ_1_-AR was inhibited by a peptide based on the turkey, but not the human, ECL2 sequence. Studies with [^3^H]-CGP 12177 demonstrated that mAb3 prevented the binding of orthosteric ligands to a subset (*circa* 40%) of turkey β_1_-receptors expressed in both CHO K1 and HEK 293 cells. MAb3 significantly reduced the maximum specific binding capacity of [^3^H]-CGP-12177 without influencing its binding affinity. Substitution of ECL2 of tβ_1_-AR with its human equivalent, or mutation of residues D186S, P187D, Q188E prevented the inhibition of [^3^H]-CGP 12177 binding by mAb3. MAb3 also elicited a negative allosteric effect on agonist-stimulated cAMP responses. The identity of the subset of turkey β_1_-adrenoceptors influenced by mAb3 remains to be established but mAb3 should become an important tool to investigate the nature of β_1_-AR conformational states and oligomeric complexes.

## Graphical abstract

**Figure:**
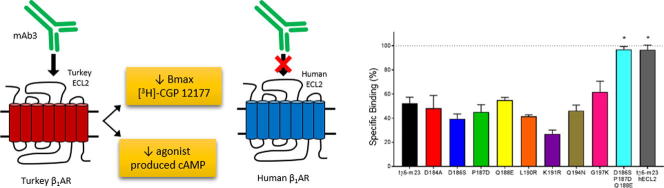

## 1 Introduction

G protein-coupled receptors (GPCRs) represent the largest class of cell surface receptors in the human genome, and are the primary target for approximately 30% of currently marketed (mostly small molecule) drugs [1], [2]. In recent years, however, there has been renewed interest in the potential development of therapeutic antibodies for this superfamily of cell surface receptors [3], [4], [5]. GPCRs consist of seven transmembrane-spanning α-helices (TM1-7), an extracellular N-terminus, an intracellular C-terminus, three extracellular loops (ECL1-3) and three intracellular loops (ICL1-3). The extracellular regions have the greatest diversity between different GPCRs [6], even between closely related receptor subtypes such as the β_1_- and β_2_-adrenoceptors, and are attractive immunogenic targets [7], [8], [9].

Epitope mapping and functional characterisation of GPCRs have demonstrated that a major immunogenic epitope is located on the second extracellular loop (ECL2) [10], [11], [12]. The structure of the ECL2 varies greatly between different GPCRs [7], [8], [13], [14], [15], [16]. It has been suggested to have roles in ligand recognition and selectivity for the β-adrenoceptors [17], [18], [19], and has recently been implicated as a site of allosteric modulation for the adenosine A_1_ receptor [20], [21]. The structure and position of the ECL2 is critical for receptor conformation and activation [22], [23]. Most interestingly, pathogenic antibodies have been identified, that can activate the β_1_-adrenoceptor by binding to ECL2, and lead to the development of a form of cardiomyopathy [10], [24]. Furthermore, different populations of antibodies directed at ECL2, isolated from patients, have been reported to stabilise different β_1_-adrenoceptor conformations, affecting agonist-driven activation and subsequent receptor desensitisation [25].

Recent advances in crystallisation techniques for GPCRs (such as thermo-stabilised receptors: StaRs; [26], [27]) have facilitated the large-scale purification of stable GPCRs in a conformation appropriate for both X-ray crystallography and their use as antigens for monoclonal antibody generation. Previously, Hutchings et al. [28] characterised a series of monoclonal antibodies (mAbs) that were raised against the thermo-stabilised turkey β_1_-adrenoceptor (β_1_AR-m23 StaR) [8]. These were found to bind to the β_1_AR-m23 StaR [8] and were able to produce small cAMP agonist responses in CHO cells transiently expressing the wild-type turkey β_1_-adrenoceptor (tβ_1_-AR) [28]. One mAb (mAb3) was suggested to bind to an allosteric site on the tβ_1_-AR, since there were indications of non-competitive antagonism with the orthosteric antagonist dihydroalprenolol [28]. The aim of this study was to investigate in more detail the pharmacology of mAb3 at the human and turkey β_1_-adrenoceptors in cell lines stably expressing each of these receptors.

## 2 Materials and methods

### 2.1 Materials

[^3^H]-adenine, [^3^H]-CGP 12177, [^14^C]-cAMP, Microscint-20 and Ultima Gold Scintillation fluid were from Perkin Elmer (Coventry, West Midlands, UK). CGP 12177, CGP 20712A, cimaterol and ICI 118551 were from Tocris Bioscience (Bristol, UK). Decyl-β-D-maltopyranoside was from Anatrace (Berkshire, UK). Purified β_1_-m23-StaR [8] was provided by Heptares Therapeutics. The following antibodies were used: goat-anti-mouse-Rhodamine secondary antibody (Molecular Probes, Life Technologies, Paisley, UK), horse radish peroxidase-conjugated secondary antibody (Cell Signalling Technology, Leiden, The Netherlands). BODIPY-TMR-CGP (CGP-12177-TMR) was purchased from Molecular Probes (Eugene, OR, USA). Fugene HD transfection reagent and furimazine were from Promega (Southampton, UK). Purified turkey and human ECL2 peptides were obtained from Cambridge Research Biochemicals (Cambridge, UK). All other reagents were from Sigma-Aldrich (Gillingham, UK).

### 2.2 Constructs, cell lines and cell culture

To create the NL-tβ6-m23 construct, NanoLuc (NL) was ligated into pcDNA3.1 containing the 5-HT_3A_ membrane localisation signal sequence (sig) as previously described [29]. The tβ6-m23 receptor was then ligated to the C-terminus of NL from pcDNA3.1 with tβ6-m23 [30] using BamHI and XbaI restriction enzymes. Mutagenic primers were then used to mutate the start codon of the tβ6-m23 receptor (Met into Leu) to create a fusion protein with a Gly-Ser linker between the NL open reading frame and the tβ6-m23 open reading frame. Finally, to ensure there were no mismatches in the plasmid potentially introduced from the mutagenesis reaction, the NL-tβ6-m23 fusion protein was ligated into a fresh pcDNA3.1 plasmid using KpnI and XbaI restriction enzymes. The ECL2 point mutations for tβ6-m23 were generated using QuickChange mutagenesis protocol (Stratagene, La Jolla, CA, USA) see Table 1 for primer sequences. The triple ECL2 mutant of D186S, P187D, Q188E was generated in two stages by sequential point mutations. First, the single D186S mutation was made as described above. Then, the triple mutant of D186S + P187D + Q188E was made with primers in Table 1 using the single D186S mutant sequence as the PCR template. All mutation sequences were confirmed by DNA sequencing using the School of Life Sciences Sequencing Facility at the University of Nottingham. Following successful mutagenesis, the mutant tβ6-m23 receptor cDNA was digested with BamHI/XhoI and subcloned into native pcDNA3.1(+) containing a neomycin selection marker.

**Table 1:** Primers used to generate ECL2 tβ6-m23 mutations. Complementary oligonucleotide primers (5′ to 3′) used for site-directed mutagenesis containing the relevant mutations were as described below. Lower case letters show the changed nucleotides. Mutations were designed to convert turkey β1AR amino acids to their human equivalents. Levels of specific ligand binding (fmol/mg protein) obtained with each construct in CHO cells in the presence of 0.5–2.5 nM [^3^H]-CGP 12177 are also shown.

Mutation	Forward and reverse oligonucleotide primers (5′ to 3′)	Receptor expression level (fmol/mg)
D184A	CACTGGTGGCGGGcCGAGGACCCTCAG and CTGAGGGTCCTCGgCCCGCCACCAGTG	343 ± 40
D186S	GTGGCGGGACGAGtcCCCTCAGGCGCTC and GAGCGCCTGAGGGgaCTCGTCCCGCCAC	264 ± 50
P187D	GCGGGACGAGGACgaTCAGGCGCTCAAG and CTTGAGCGCCTGAtcGTCCTCGTCCCGC	237 ± 68
Q188E	GGACGAGGACCCTgAGGCGCTCAAGTG and CACTTGAGCGCCTcAGGGTCCTCGTCC	359 ± 92
L190R	GAGGACCCTCAGGCGCgCAAGTGCTACCAGGACCCG and CGGGTCCTGGTAGCACTTGcGCGCCTGAGGGTCCTC	466 ± 10
K191R	GAGGACCCTCAGGCGCTCAgGTGCTACCAGGACCCG and CGGGTCCTGGTAGCACcTGAGCGCCTGAGGGTCCTC	659 ± 67
Q194N	GCGCTCAAGTGCTACaatGACCCGGGCTGCTGC and GCAGCAGCCCGGGTCattGTAGCACTTGAGCGC	218 ± 50
G197K	GTGCTACCAGGACCCGaaaTGCTGCGACTTTGTCAC and GTGACAAAGTCGCAGCAtttCGGGTCCTGGTAGCAC	290 ± 63
D186S + P187D + Q188E	GCGGGACGAGTCCgaTgAGGCGCTCAAG and CTTGAGCGCCTcAtcGGACTCGTCCCGC	671 ± 31

Chinese hamster ovary (CHO-K1) cells stably expressing either the human β_1_-adrenoceptor, the C-terminally truncated turkey β-adrenoceptor (tβtrunc; [31]), or an N-terminally truncated tβtrunc with the m23 thermostabilising mutations (tβ6-m23; [30], [31]) were used in this study. In addition to the β-adrenoceptor, these CHO-K1 cells also stably expressed a cyclic AMP response element-secreted placental alkaline phosphatase (CRE-SPAP) reporter gene. HEK 293 cells transiently expressing NL-tβ6-m23 were also used in this study. Cells were cultured in Dulbecco’s modified Eagle’s medium nutrient mix F12 (DMEM/F12, CHO cells) or Dulbecco’s modified Eagle’s medium (DMEM, HEK cells) containing 10% foetal calf serum and 2 mM L-glutamine in a 37 °C humidified 95% air/5% CO_2_ atmosphere. All CHO cell lines were kindly provided by Prof. Jill Baker.

### 2.3 Enzyme-linked Immunosorbant assays (ELISAs)

Purified β_1_AR-m23 StaR, [8] was immobilised through the capture of the C-terminal 6-His tag to Ni^+^-coated Nunc Immobilizer 96-well plates. Each well was coated with PBS containing 0.5 μg/ml purified protein solubilised in 0.1% DM (n-Decyl-β-maltopyranoside) and 100 μM alprenolol. Each well was blocked with PBS containing 0.1% DM, 100 μM alprenolol and 3% milk powder (blocking solution) for 1 h at room temperature. During this stage, dilutions of mAb3 were incubated with 250 μg/ml or 500 μg/ml ECL2 peptide in blocking solution for 1 h (Human ECL2 peptide: MHWWRAESDEARRCYNDPKCCDFVTN, Turkey ECL2 peptide: MHWWRDEDPQALKCYQDPGCCDFVTN; Cambridge Research Biochemicals, Cambridge, UK). Following this, each well was incubated with the mAb3/ECL2 blocking solution for 1 h. Wells were then washed with 3 × 200 μl of PBS with 0.05% Tween-20, 0.1% DM and 100 μM alprenolol. A goat anti-mouse horseradish peroxidase (HRP)-conjugated secondary antibody (1:1000) was added to each well in 100 μl PBS with 0.05% tween-20, 0.1% DM and 100 μM alprenolol. Bound antibody was detected using the 3,3′,5,5′-Tetramethylbenzidine (TMB) liquid substrate system (Sigma-Aldrich, Gillingham, UK). The absorbance was measured at 655 nm using a POLARstar Omega plate reader (BMG Labtech, Germany).

### 2.4 Wide-field microscopy

Wide-field microscopy was performed using an ImageXpress Micro XLS System microscope with a Plan Fluor ELWD 20× objective and a 4.66 megapixel CMOS camera for the acquisition of images. CHO cells were grown to confluence in sterile black-sided tissue culture-treated 96-well plates. For ECL2 competition assays, cells were fixed with 3% paraformaldehyde in PBS for 15 min at room temperature. Cells were washed three times with ice-cold PBS and blocked with 0.5% bovine serum albumin (BSA) in PBS (blocking solution) for 30 min on a shaker (60 rpm) at 4 °C. During this period, 66 nM mAb3 was incubated with the purified turkey or human ECL2 peptide in the blocking solution. The blocking solution was removed from the cells and replaced with the mAb3/ECL2 solution for 1 h at room temperature.

Cells were then washed three times with ice-cold PBS and then incubated with the secondary antibody (goat anti-mouse-Rhodamine Red IgG, 1:500 in 10% goat serum with PBS) for 1 h in the dark at room temperature. This was then washed with three ice-cold PBS washes before cells were stained with 2 μg/ml Hoechst 33342 stain in PBS for 20 min. Three cold PBS washes removed any excess stain and the plates were stored at 4 °C in the dark overnight before being imaged on the ImageXpress Micro XLS System microscope (Molecular Devices, Wokingham, UK). Each site was imaged twice, once with the ZPS DAPI filter (ex 405 nm) to capture the Hoechst 33342 nuclear stain, and again with the ZPS CY5/TRITC filter (ex 560 nm) to capture the Rhodamine from the secondary antibody.

### 2.5 [^3^H]-CGP 12177 radioligand binding

All experiments were performed in Dulbecco’s modified Eagle’s medium mix F12 containing 2 mM L-glutamine (serum-free media). CHO cells were grown to confluence in sterile white-sided, tissue culture-treated 96-well plates. For competition binding experiments, the media were aspirated from each well and replaced with 50 μl serum-free media containing [^3^H]-CGP 12177 within the range of 0.8–2.5 nM. Competing drugs were added in 50 μl serum-free media to ensure thorough mixing and the plates incubated at 37 °C for 2 h. Non-specific binding was determined with 10 μM propranolol. The serum-free media were then removed and each well was washed twice with 200 μl cold PBS. 100 μl Microscint-20 fluid was then added to each well and the plates were sealed and left for several hours at room temperature before being counted on a Topcount for 2 min per well. For saturation experiments [^3^H]-CGP 12177 was used in concentrations ranging from 0.01 to 40 nM. 10 μM propranolol was used to define non-specific binding. When measuring allosterism, mAb3 was allowed to bind to the tβ6-m23 receptor for 30 min at 37 °C before the competing ligands or [^3^H]-CGP 12177 was added. The protein content of each well was determined as described previously [32].

### 2.6 NanoBRET ligand binding assay

Saturation- and competition-binding assays were performed on transiently transfected HEK 293 NL-tβ6-m23 cells. White 96-well microplates were coated with 50 µl poly-D-lysine (10 µg/ml in PBS) for 30 min and then washed with 100 µl PBS. Following this, 5 µl transfection mix (Optimem with 100 ng NL-tβ6-m23 plasmid DNA and 50 µl/ml Fugene HD transfection reagent) was added to each well. 100 µl DMEM with cells in suspension (20,000 cells/well) was then seeded onto these wells containing the transfection mix to ensure complete mixing of the transfection mix and cells. After 24 h, the medium was removed from each well and replaced with HEPES-buffered saline solution (HBSS; 145 mM NaCL, 5 mM KCl, 1.7 mM CaCl_2_, 1 mM MgSO_4_, 10 mM HEPES, 2 mM sodium pyruvate, 1.5 mM NaHCO_3_, 10 mM D-glucose, pH 7.45) with the relevant concentration of fluorescent ligand and, if necessary, competing ligand or mAb3. Nonspecific binding was determined with 10 µM propranolol. Cells were then incubated for 1 h at 37 °C in the dark. The NanoLuc substrate furimazine (Promega, Southampton, UK) was then added to each well at a final concentration of 10 µM and allowed to equilibrate for 5 min prior to reading. Luminescence signals were measured at two different wavelengths using a PHERAstar FS plate reader (BMG Labtech, UK) at room temperature. The filtered light from each well was simultaneously measured using 460 nm (80-nm bandpass) and >610 nm longpass filters. The resulting raw BRET ratio was calculated by dividing the >610 nm emission by the 460 nm emission.

For saturation NanoBRET experiments with mAb3, HEK cells transiently expressing NL-tβ6-m23 were fixed with 3% paraformaldehyde in PBS for 15 min at room temperature. Cells were then washed three times with PBS before being incubated with 100 µl PBS containing mAb3 in a concentration range of 1–250 nM for 1 h at 37 °C. The cells were then washed three times with PBS and then incubated with the secondary antibody (goat anti-mouse-Rhodamine Red IgG, 1:500 in 10% goat serum with PBS) for 1 h in the dark at room temperature. Finally, furimazine was added and the BRET was measured as described above.

### 2.7 [^3^H]-cAMP accumulation

CHO cells stably expressing the human or turkey β-adrenoceptors were grown to confluence in sterile, clear, tissue culture-treated 48-well plates. Cells were pre-labelled with [^3^H]-adenine for 2 h with 2 μCi/ml [^3^H]-adenine at 37 °C. Cells were then washed by the addition and removal of 500 μl serum-free media before the addition of 225 μl serum-free media. Where used, 1 mM IBMX (3-isobutyl-1-methylxanine) was added and incubated for 30 min before the addition of ligands. Ligands were added in 25 μl serum-free media to the relevant wells and the plates were incubated at 37 °C for up to 5 h. 10 μM isoprenaline was used to determine the maximal response in each plate for each experiment. Where total [^3^H]-cAMP was measured, the assay was terminated by the addition of 50 μl 12 M HCl to each well and the plates were then frozen. Plates were subsequently thawed and [^3^H]-cAMP separated from other nucleotides by sequential AG 50W-4X resin and alumina column chromatography, using [^14^C]-cAMP to define column efficiency (as described in [33]). Extracellular and intracellular [^3^H]-cAMP were measured separately where stated. Here, the extracellular media were removed after the given ligand incubation time and transferred to a separate 48-well plate for the measurement of extracellular [^3^H]-cAMP. The cells were washed by the addition and removal of 2 × 500 μl serum-free media. 250 μl serum-free media was then added to each well and the reaction terminated by the addition of 50 μl 12 M HCl to all wells (including those on the extracellular [^3^H]-cAMP plate). Column separation of [^3^H]-cAMP from other nucleotides was then performed as previously described in [34].

### 2.8 CRE-mediated gene transcription (SPAP)

CHO cells stably expressing the CRE-SPAP reporter gene and either human or turkey β-adrenoceptors were grown to confluence in sterile, clear, tissue culture-treated 96-well plates and serum-starved 24 h before experimentation in serum-free media. On the day of experimentation, media were aspirated from each well and replaced with 90 μl fresh serum-free media. Ligands were then added (in triplicate) in 10 μl serum-free media and the plate was incubated at 37 °C in a humidified 95% air/5% CO_2_ atmosphere for 5 h. 10 μM isoprenaline was used to determine the maximal response in each plate for each experiment. Media and compounds were then aspirated and replaced with 40 μl serum-free media. Plates were incubated for a further hour at 37 °C in the same atmosphere. The plates were then heated to 65 °C for 25 min to destroy any endogenous alkaline phosphatases. After allowing them to cool to room temperature, 100 μl diethanolamine buffer (pH 9.85) containing p-nitrophenol phosphate (PNPP) was added to each well and the plates incubated at 37 °C for 20 min in air. CRE-mediated SPAP reporter activity was quantified by the colour change resulting from the hydrolysis of PNPP. This was measured as the optical density at 405 nm using a Dynatech Laboratories MRX plate reader.

### 2.9 Data analysis

Data were analysed using Prism 6 software (GraphPad, San Diego, USA).

Saturation radioligand-binding curves were fitted simultaneously for total ([^3^H]-CGP 12177 alone) and non-specific binding (in the presence of 10 µM propranolol) using the following equation:

$$\mathrm{Total}\;\mathrm{binding}=\frac{B_{\mathrm{MAX}}\times [B]}{[B]+K_{D}}+m\times [B]+C$$

where *B_max_* is the maximal specific binding, [B] the concentration of fluorescent ligand (nM), *K_D_* the equilibrium dissociation constant (nM), *M* the slope of the non-specific binding component and *C* the *y* axis intercept.

The affinity of CGP 20712A was calculated from radioligand binding data with a one-site sigmoidal response curve given by the following equation:

$$\%\;\mathrm{uninhibited}\;\mathrm{binding}=100-\frac{\left(100\times [A^{n}]\right)}{\left([A^{n}]+{\mathrm{IC}}_{50}^{n}\right)}+\mathrm{NS}$$

where [A] is the concentration of CGP 20712A, NS is nonspecific binding, *n* is the Hill coefficient, and IC_50_ is the concentration of ligand required to inhibit 50% of the specific binding of [^3^H]-CGP 12177. The IC_50_ values were then used to calculate the K_i_ of CGP 20712A using the Cheng-Prussoff equation:

$$K_{i}=\frac{{\mathrm{IC}}_{50}}{1+\frac{\left[L\right]}{K_{D}}}$$

where [L] is the concentration of [^3^H]-CGP 12177 in nM, and K_D_ is the dissociation constant of [^3^H]-CGP 12177 in nM.

The affinity of mAb3 was calculated from radioligand binding data following the allosteric ternary complex model previously described [35]:

$${\mathrm{IC}}_{50}=K_{B}\left(\frac{[A]+K_{A}}{\alpha [A]+K_{A}}\right)$$

where IC_50_ is the concentration of allosteric modulator (B) required for 50% inhibition of specific binding of a fixed concentration [A] of radioligand, K_A_ and K_B_ are the dissociation constants for the radioligand and allosteric modulator respectively, and α is the cooperativity factor between the allosteric modulator and radioligand.

Specific binding curves for CGP 12177 or mAb3 were fit to the following equation:

$$\mathrm{Specific}\;\mathrm{Binding}=\frac{B_{\mathrm{MAX}}\times [B]}{[B]+K_{D}}$$

where *B_max_* is the maximal specific binding, [B] the concentration of fluorescent ligand or mAb3 (nM) and *K_D_* the equilibrium dissociation constant (nM).

Agonist response curves were best described by a one-site sigmoidal concentration response curve using the following equation:

$$\mathrm{Response}=\frac{E_{\max}\times [A]}{{\mathrm{EC}}_{50}+[A]}$$

where E_max_ is the maximum system response, [A] is the agonist concentration, and EC_50_ is the concentration of agonist required to produce 50% of the maximal response. The affinity of mAb3 was estimated from [^3^H]-cAMP accumulation and CRE-SPAP reporter gene assays using the model described in [36].

The relative activity (RA) was first calculated for isoprenaline in the presence of each concentration of mAb3 using the following equation:

$$\mathrm{RA}=\frac{E_{\max}.{\mathrm{EC}}_{50}^{\prime }}{E_{\max}^{\prime }{\mathrm{EC}}_{50}}$$

where RA is the relative activity of the agonist in the presence of the allosteric modulator, E_max_′ and EC_50_′ denote the E_max_ and EC_50_ values of the agonist in the absence of the allosteric modulator, and E_max_ and EC_50_ are the values measured of the agonist in the presence of the allosteric modulator. This analysis was performed with the constraint that the Hill slopes were equal to a value of 1.

To obtain an estimate for the affinity of mAb3, the RA obtained at each concentration of mAb3 was plotted against the concentration of mAb3 (B). Nonlinear regression analysis of these data was performed using the following equation in Prism 6:

$$\log\mathrm{RA}=\log\left(\frac{1+\gamma [B]/K_{B}}{1+[B]/K_{B}}\right)$$

where K_B_ is the dissociation constant of mAb3 (B), and γ is the product of the effects the mAb3 has on the intrinsic efficacy and affinity of the agonist-receptor complex.

The time course of the [^3^H]-cAMP accumulation in response to 10 μM isoprenaline in tβ6-m23 cells was fitted using the following equation:

$$Y=Y_{\max}(1-e^{-k_{\mathrm{obs}}.t})$$

where

*k_obs_* = ([^3^H]-cAMP x *k_on_*) + *k_off_*.
*k_on_* and *k_off_* were shared across the data set to obtain a single value for *k_obs_*. Y was [^3^H]-cAMP (dpm) at time t (min) and Y_max_ was the maximum accumulation of [^3^H]-cAMP induced by isoprenaline.

Inhibition of the steady-state levels of intracellular [^3^H]-cAMP (produced by isoprenaline) following addition of mAb3 and CGP 20712A tβ6-m23 cells were fitted using a one-phase exponential decay equation:

$$Y={\left(Y_{0}-\mathrm{NS}\right)}^{-k.t}+\mathrm{NS}$$

where Y_0_ represented the intracellular [^3^H]-cAMP at time 0, NS is the intracellular [^3^H]-cAMP at infinite time, and *k* is the rate constant of the decrease in intracellular [^3^H]-cAMP per minute.

Statistical significance was determined as p < 0.05 with paired and unpaired *t*-tests, as well as one-way analysis of variance (ANOVA). Goodness of statistical fits were analysed by a Partial F test (Prism 6).

## 3 Results

### 3.1 MAb3 binding to ECL2 of the purified turkey β_1_AR-m23 StaR receptor

**Fig. 1:**
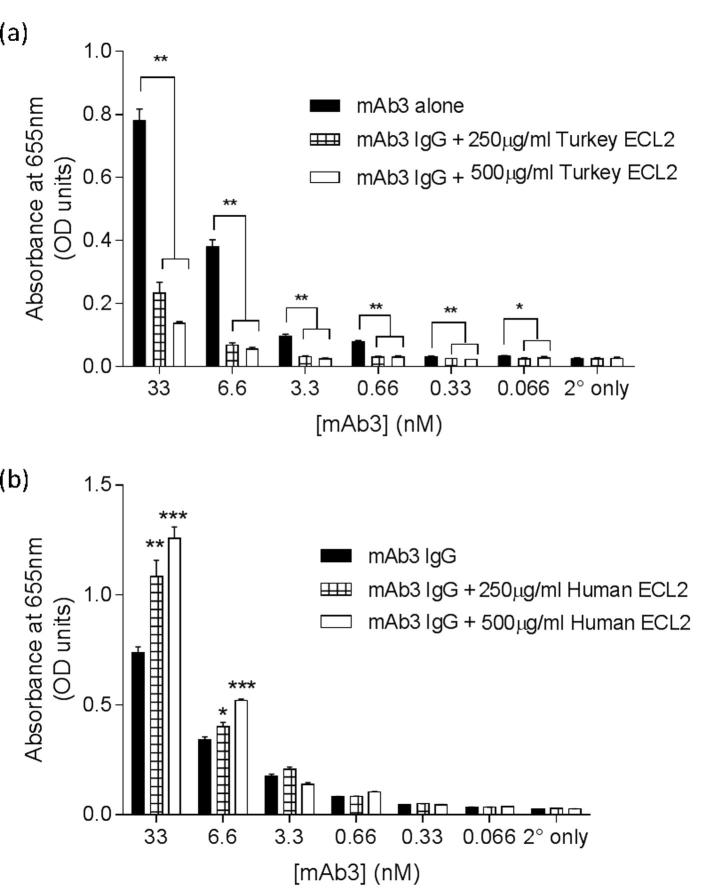
ELISA showing the effect of a (a) turkey or (b) human ECL2 peptide sequence (250 μg/ml or 500 μg/ml) on the binding of different concentrations of mAb3 to purified β_1_AR-m23 StaR. Included are controls for the secondary antibody alone, and the secondary antibody in combination with the relevant peptide. Bars are mean ± s.e.m. of triplicate determinations and these single experiments are representative of four separate experiments. ^*^p < 0.05, ^**^p < 0.01, ^***^p < 0.001, comparing binding of mAb3 at β_1_AR-m23 StaR to the other conditions, one-way ANOVA with Dunnett’s post hoc test.

The crystal structure of the β_1_-adrenoceptor was first solved using a thermo-stabilised turkey β_1_-AR (β_1_AR-m23 StaR) [8]. This construct and the resulting protein produced were used to generate a panel of monoclonal antibodies using a combination of cDNA immunization and protein boosting [28]. One of these antibodies (mAb3) was demonstrated to bind to the β_1_AR-m23 StaR expressed in HEK cells. Using purified proteins, the epitope of mAb3 was suggested to be located on the second extracellular loop (ECL2) of the turkey β_1_-AR [28]. Here, we investigated the effect of a purified ECL2 peptide on the binding of mAb3 to the β_1_AR-m23 StaR. Pre-incubation of mAb3 with purified turkey β_1_-AR ECL2 (Tk ECL2) significantly reduced the specific binding of mAb3 to the purified β_1_AR-m23 StaR in a concentration-dependent manner (Fig. 1a, p < 0.05, one way ANOVA). In contrast, pre-incubation of mAb3 with purified human β_1_-AR ECL2 (Hu ECL2) did not significantly attenuate binding to β_1_AR-m23 StaR, but rather produced a significant increase in specific binding at the highest concentrations of mAb3 used (33 nM and 6.6 nM; Fig. 1b, p < 0.05, one way ANOVA).

### 3.2 Immunohistochemistry of mAb3 binding to CHO cells expressing β_1_-ARs

With mAb3 showing strong binding to the purified β_1_AR-m23 StaR, we next investigated the binding capabilities of mAb3 in CHO cells. Two CHO cell lines stably expressing the turkey β_1_-AR were used in this study: a low expressing cell line (tβtrunc, receptor expression level of 92 ± 14 fmol/mg protein, n = 8; determined in this study with [^3^H]-CGP 12177 binding; [37]), and a high expressing cell line (tβ6-m23, receptor expression level 3128 ± 590 fmol/mg protein, n = 8; [30]). In addition, a CHO cell line stably expressing the human β_1_-AR (hβ_1_-AR) was used (receptor expression level 659 ± 77 fmol/mg protein, n = 6; [38]).

**Fig. 2:**
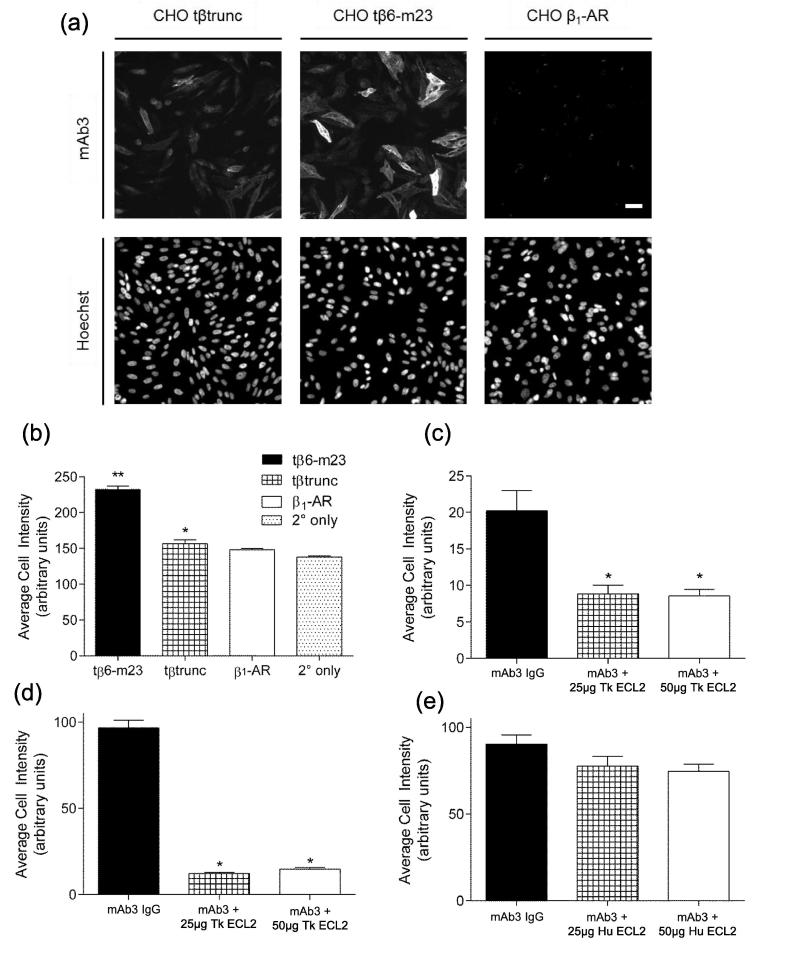
(a) Wide-field microscopy of mAb3 binding to fixed CHO cells expressing tβtrunc, tβ6-m23 or hβ_1_-AR. Cells were treated with 10 μg/ml (66 nM) mAb3 for one hour at room temperature. Images show the localisation of mAb3 staining (upper panels) and Hoechst 33342 nuclear stain (lower panels). Scale bar is 20 μm and applies to all panels. Images were obtained from a single experiment and this is representative of four separate experiments. (b) Average cell intensity of images shown in (a) in greyscale units. Specific binding of mAb3 at (c) tβtrunc, (d-e) tβ6-m23 in the absence and presence of 25 μg or 50 μg turkey ECL2 (Tk ECL2, c-d) or 25 μg or 50 μg human ECL2 (Hu ECL2, e). (c–e) values are specific mAb3 binding, as the intensity of 2° only has been subtracted. (b–e) Data points are mean ± s.e.m. from triplicate determinations in a single experiment. These single experiments are representative of four separate experiments. ^*^p < 0.01, ^**^p < 0.001, comparing (b) average cell intensity at tβtrunc and tβ6-m23 to the 2° only control or (c–d) the average cell intensity of mAb3 binding in the presence of TkECL2 to that of Mab3 IgG alone, one-way ANOVA.

The specific binding of mAb3 to CHO cells expressing β_1_-ARs was visualised using immunocytochemistry (Fig. 2). MAb3 was able to specifically label the turkey β_1_-AR stably expressed on the surface of intact CHO cells (Fig. 2a). CHO cells stably expressing the human β_1_-AR (hβ_1_-AR) did not display any binding of mAb3 (Fig. 2a). Quantitative analysis on these images calculated the average fluorescence intensity of cells as a measure of mAb3 binding. The average cell intensity of the secondary antibody-only controls was subtracted from the intensities of cells treated with mAb3 to give a measure of the specific intensity of mAb3 binding (corrected cell intensity). CHO cells expressing the tβtrunc and tβ6-m23 receptors had significantly higher average cell intensities than the secondary antibody-only control (corrected average cell intensities tβ6-m23 55.0 ± 8.5 greyscale units; tβtrunc 15.3 ± 2.9 greyscale units; n = 4, Fig. 2b, p < 0.05, paired *t*-test). The relative degree of mAb3 labelling was consistent with the relative receptor expression levels calculated using radioligand binding. This analysis also confirmed there was no specific binding of mAb3 to the hβ_1_-AR, since the average cell intensity did not significantly differ from the secondary antibody-only control (corrected cell intensity hβ_1_-AR 2.3 ± 1.1 greyscale units; n = 4, Fig. 2b, p > 0.05, paired *t*-test).

The specific binding of mAb3 to the turkey receptors was displaced by addition of either 250 μg/ml or 500 μg/ml purified Tk ECL2 peptide (Fig. 2c, d). Pre-incubation of mAb3 with Tk ECL2 at both concentrations reduced specific binding to the tβtrunc receptor to *circa* 50% that of mAb3 alone (250 μg/ml Tk ECL2, 49.0 ± 9.9%; 500 μg/ml Tk ECL2, 47.9 ± 5.3%, respectively; n = 4). This effect was more pronounced in cells expressing the tβ6-m23 receptor. In these cells, the addition of Tk ECL2 resulted in only 31.2 ± 10.6% (250 μg/ml Tk ECL2) and 21.7 ± 6.6% (500 μg/ml Tk ECL2) of the specific binding elicited by mAb3 alone (n = 4, Fig. 2d). Interestingly, pre-incubation of mAb3 with Hu ECL2 peptide did not result in a significant reduction of mAb3 binding to the tβtrunc or tβ6-m23 receptors (500 µg/ml 76.8 ± 32.6% tβtrunc; 88.0 ± 5.6% tβ6-m23, Fig. 2e, p > 0.05 paired *t*-test).

### 3.3 Effect of mAb3 on the specific binding of [^3^H]-CGP 12177 to turkey β_1_-adrenoceptors stably expressed in CHO cells

**Fig. 3:**
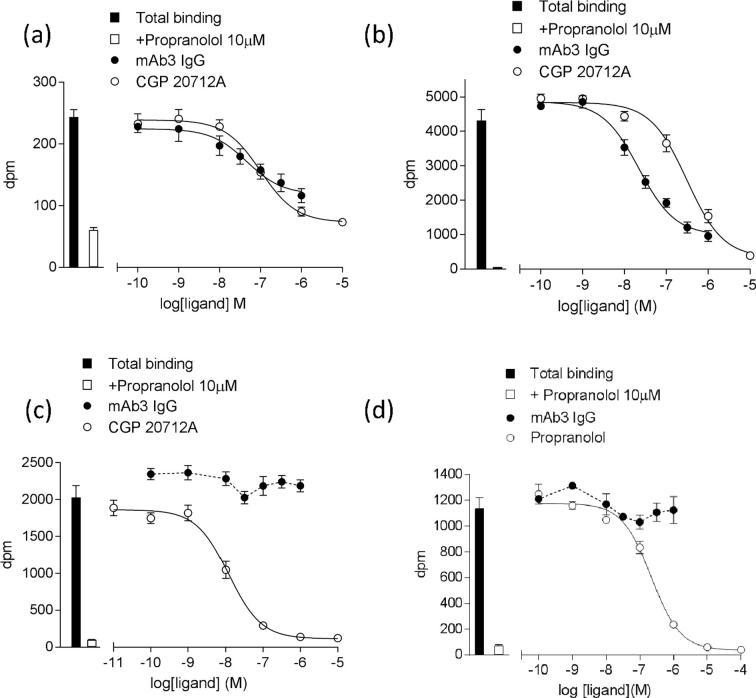
[^3^H]-CGP 12177 radioligand binding in whole CHO cells expressing (a) tβtrunc, (b) tβ6-m23 (c) hβ_1_-AR or (d) tβ6-m23-hECL2 in the presence of increasing concentrations of (a-c) mAb3, CGP 20712A, or (d) mAb3, propranolol. The concentration of [^3^H]-CGP 12177 used was (a) 0.56 nM, (b) 0.67 nM (c) 0.56 nM and (d) 0.76 nM. Bars indicate total [^3^H]-CGP 12177 binding and [^3^H]-CGP 12177 binding in the presence of 10 μM propranolol. Data are mean ± s.e.m. of (a-c) hexuplicate or (d) triplicate determinations in a single experiment. These single experiments are representative of (a) seven, (b) seven (c) eight or (d) five separate experiments.

The radioligand [^3^H]-CGP 12177 was able to specifically bind to the three β_1_-ARs used in this study to give K_D_ values of 1.67 ± 0.41 nM (n = 7) for the tβtrunc, 0.93 ± 0.19 nM (n = 7) for the tβ6-m23, and 0.75 ± 0.13 (n = 13) for the hβ_1_-AR. The β_1_-selective antagonist CGP 20712A was able to compete with the specific binding of [^3^H]-CGP 12177 in all three β_1_-ARs, yielding log K_i_ values of −7.23 ± 0.09 (n = 7) for the tβtrunc, −6.88 ± 0.13 (n = 7) for the tβ6-m23, and −8.70 ± 0.08 (n = 8) at the hβ_1_-AR, respectively. Increasing concentrations of mAb3 were also able to inhibit the specific binding of [^3^H]-CGP 12177 to both the tβtrunc and tβ6-m23 receptors (Fig. 3a and b). However, the maximal inhibition of specific [^3^H]-CGP 12177 binding by mAb3 was lower than that produced by the orthosteric antagonist CGP 20712A in the same experiments (Fig. 3a and b, Table 2). Taken together with the fact that the epitope of mAb3 was thought to be on ECL2 [28], these data suggested that mAb3 may be acting allosterically with respect to the known small molecule orthosteric binding site on the turkey β_1_-ARs. Statistical comparison was performed to ascertain whether the allosteric ternary complex model (ATCM, [35]) was the best model to describe these data. For both receptors, globally analysing the data with the ATCM provided a better description of the data than the standard three-parameter inhibition model (p < 0.001, tβtrunc; p < 0.05 tβ6-m23, partial F test). Application of the ATCM provided estimates for the affinity of mAb3 at the tβtrunc and tβ6-m23 receptors, as well as an estimate for the cooperativity factor (α) between mAb3 and [^3^H]-CGP 12177 (Table 2). From these analyses, mAb3 had similar affinities at the tβtrunc (log K_B_ −7.56 ± 0.08) and at the tβ6-m23 (log K_B_ −7.70 ± 0.09). The estimated α values at both tβtrunc and tβ6-m23 suggested a high degree of negative cooperativity between mAb3 and [^3^H]-CGP 12177 at these receptors. There was no evidence of mAb3 binding to the hβ_1_-AR (Fig. 3c), which was consistent with the data obtained with the purified Hu ECL2 peptide.

**Table 2:** Log IC_50_, log K_B,_ α values and % maximal inhibition of [^3^H]-CGP 12177 binding obtained from [^3^H]-CGP 12177 whole cell radioligand binding studies in CHO cells stably expressing either the tβtrunc, tβ6-m23, or hβ_1_-adrenoceptors. Maximal inhibition of specific [^3^H]-CGP 12177 binding was determined in the presence of 10 μM propranolol. Data are mean ± s.e.m. from n separate experiments. NI – No inhibition. The ATCM (allosteric ternary complex model; [35]) was performed to obtain the log K_B_ of mAb3 using a global fit. ^*^p < 0.05 comparing maximal inhibition to 100% representing complete inhibition of specific binding with 10 μM propranolol, unpaired *t*-test. ^†^p < 0.05, ^‡^p < 0.001, comparing ATCM fit to three-parameter inhibition curve, F-test.

CHO Cell Line	Log IC_50_ mAb3	% Max Inhibition of [^3^H]-CGP 12177 binding			ATCM fit		Preferred fit
		10 μM CGP 20712A	1 μM mAb3	n	Log K_B_ mAb3	mAb3 α value	Partial F-test
tβtrunc	−7.48 ± 0.12	84.3 ± 3.9^*^	63.4 ± 7.1^*^	7	−7.56 ± 0.08	0.06 ± 0.03	ATCM^‡^
tβ6-m23	−7.32 ± 0.12	94.7 ± 0.5^*^	77.5 ± 4.5^*^	7	−7.70 ± 0.09	0.05 ± 0.02	ATCM^†^
hβ_1_-AR	NI	99.6 ± 0.3	NI	8	NI	NI	–

To investigate further the role that the ECL2 plays in the binding of mAb3 to the full length receptor, a mutant tβ6-m23 receptor construct was made whereby the Tk ECL2 was mutated to the Hu ECL2 sequence (tβ6-m23-hECL2), and this construct was transiently expressed in CHO cells. Specific binding of [^3^H]-CGP 12177 was observed at this receptor (K_D_ 7.85 ± 0.88 nM, B_max_ 2857 ± 262 fmol/mg protein; n = 4), demonstrating that the resulting receptor protein was expressed on the plasma membrane. Increasing concentrations of propranolol were able to inhibit the specific binding of [^3^H]-CGP 12177 (log K_i_ propranolol −6.73 ± 0.08; n = 5). However, as with the hβ_1_-AR, mAb3 could not bind to the tβ6-m23-hECL2 receptor (Fig. 3d), further suggesting that the epitope of mAb3 existed entirely within the turkey ECL2.

**Fig. 4:**
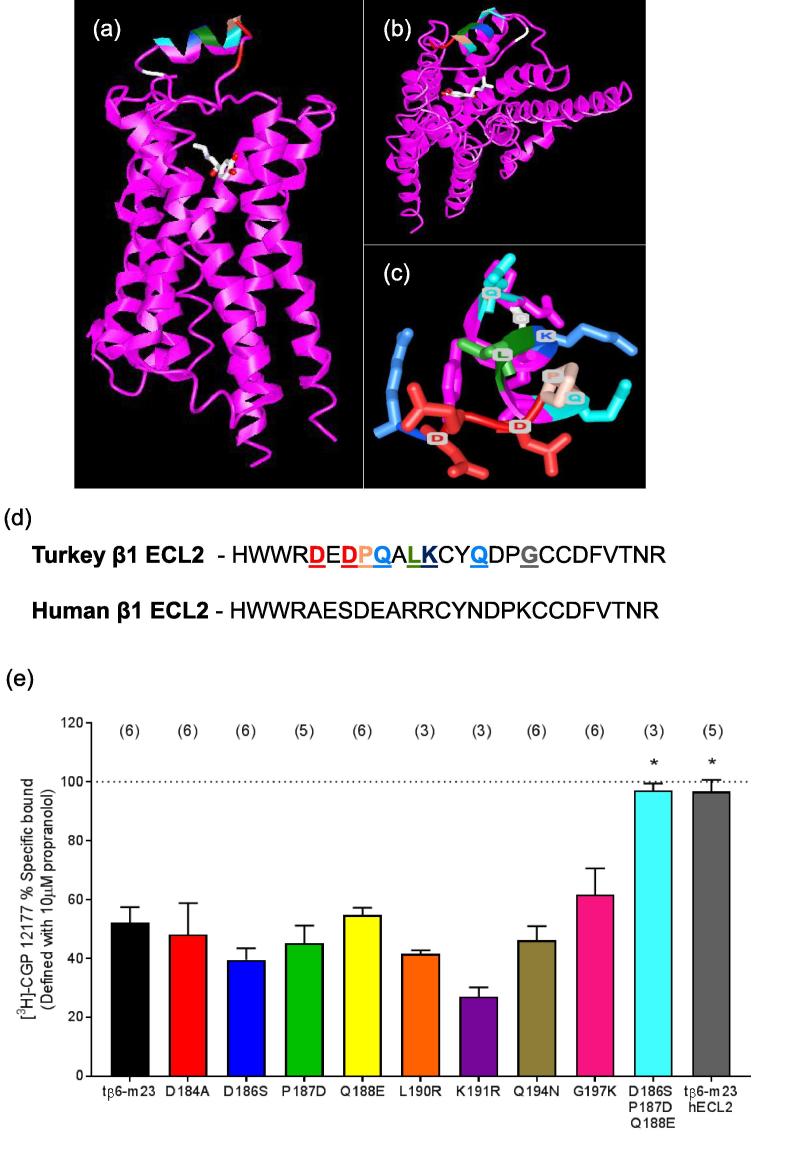
Ribbon representation of the β_1_AR-m23 StaR structure bound to isoprenaline (PDB ID: 2Y03; [39]) generated with Cn3D (National Centre for Biotechnology Information). (a) Side view and (b) top view of receptor highlighting the ECL2 residues (coloured as in (d)) which differ between the turkey and human β_1_-AR sequences. The structure of isoprenaline is also shown. (c) Top view of ECL2 with side chains of residues (coloured) that differ from the human β_1_-AR sequence. (d) Amino acid sequence of ECL2 for turkey and human β_1_-AR, with residue differences highlighted. (e) Specific binding of 0.5–2.5 nM [^3^H]-CGP 12177 in the presence of 1 µM mAb3 in CHO cells transiently expressing wildtype tβ6-m23 or ECL2 mutant constructs (where residues have been changed from the turkey amino acid to their human equivalent). Data are represented as a % of the specific binding of [^3^H]-CGP 12177 in the presence of mAb3. Non-specific binding was defined with 10 µM propranolol. Data are mean ± s.e.m. The number of separate experiments performed for each mutant are given in parentheses above each bar. Triplicate determinations were performed in each individual experiment. ^*^p < 0.05 comparing the inhibition specific binding by 1 µM mAb3 in that ECL2 mutant to the wild-type receptor, one-way ANOVA. (For interpretation of the references to colour in this figure legend, the reader is referred to the web version of this article.)

The turkey ECL2 is 26 amino acids in length and shares 70% identity with the human ECL2. The eight residues that differ between the two species have been highlighted on the recently solved β_1_AR-m23 StaR crystal structure [39] (Fig. 4a-c). The majority of these residues exist on the extracellular surface of the loop (Fig. 4a), with side chains facing away from the ligand-binding pocket, projecting into the extracellular space (Fig. 4c). In order to elucidate which residues were necessary for the binding of mAb3, a series of single point mutations were made, changing the turkey residue to the equivalent human residue. The eight mutations were as follows: D184A, D186S, P187D, Q188E, L190R, K191R, Q194N, and G197K (Fig. 4d). Similar levels of specific binding of [^3^H]-CGP 12177 were obtained with all mutant constructs (Table 1). Single point mutations did not significantly affect the binding of mAb3 (Fig. 4e), demonstrating that the epitope of mAb3 was not susceptible to the loss of a single residue. Given this fact, we then looked for clusters of amino acids that may be responsible for the mAb3 epitope. Residues D186, P187, Q188 were all located on the N-terminus of the α-helix in ECL2, and offered a potential target. Therefore, a chimeric tβ6-m23 receptor containing the triple mutation D186S, P187D and Q188E was made (Table 1). Interestingly, 1 µM mAb3 did not displace the specific binding of [^3^H]-CGP 12177 at this receptor (Fig. 4e). These data suggest that this cluster of three amino acids is necessary for mAb3 binding.

**Fig. 5:**
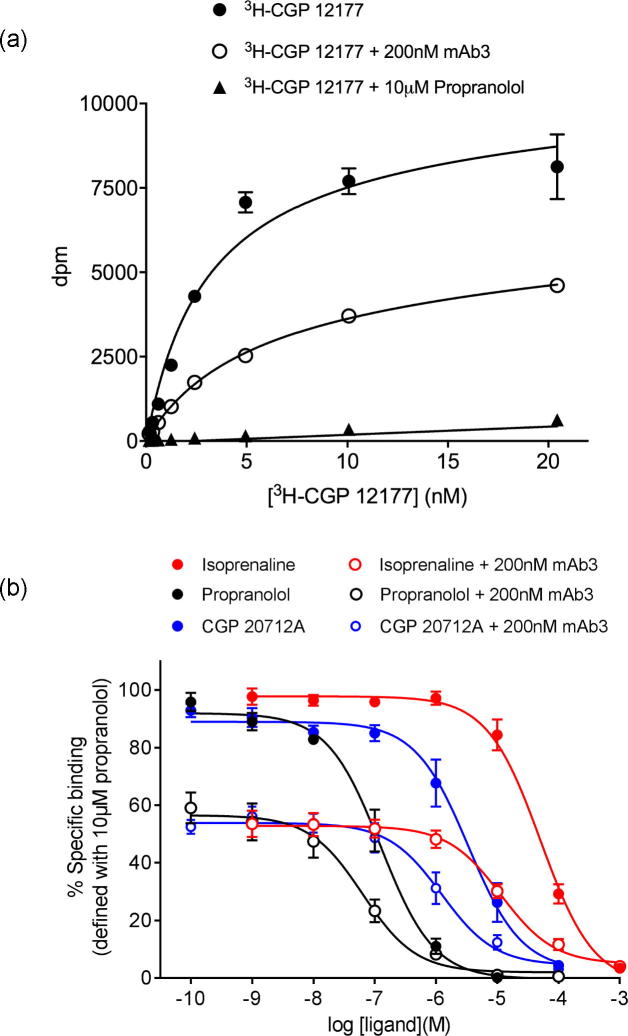
(a) [^3^H]-CGP 12177 radioligand binding in whole CHO tβ6-m23 cells in the absence and presence 200 nM mAb3. Non-specific binding was defined in the presence of 10 µM propranolol. Data points are mean ± s.e.m from triplicate determinations in a single experiment and this single experiment is representative of three separate experiments. (b) Inhibition of 0.4–1.8 nM [^3^H]-CGP 12177 binding to CHO tβ6-m23 cells by ligands in the absence and presence of 200 nM mAb3. Data points are expressed as a % of the specific binding of [^3^H]-CGP 12177 obtained in the absence of an inhibitor in each experiment. Non-specific binding was defined in the presence of 10 µM propranolol. Data points represent the mean ± s.e.m. from five separate experiments where each experiment was performed in duplicate.

The potential allosteric nature of the interaction of mAb3 with the tβ_1_-AR was further investigated using ligand binding assays. CHO tβ6-m23 cells were incubated with increasing concentrations of [^3^H]-CGP 12177 in the absence and presence of 200 nM mAb3 (Fig. 5a). In these experiments the K_D_ of [^3^H]-CGP 12177 was 2.59 ± 0.18 nM (n = 3), and the affinity of [^3^H]-CGP 12177 in the presence of 200 nM mAb3 was not significantly changed (4.13 ± 0.54 nM; n = 3, p > 0.05, paired *t*-test). However, the addition of 200 nM mAb3 resulted in a reduced B_max_ of [^3^H]-CGP 12177 (58.4 ± 13.9% total specific binding; p < 0.05 compared to specific binding in the absence of mAb3, paired *t*-test).

Competition [^3^H]-CGP 12177 binding assays with the orthosteric ligands isoprenaline, propranolol and CGP 20712A were also performed in the absence and presence of 200 nM mAb3 (Fig. 5b). The affinities of the orthosteric ligands determined here showed good agreement with previously published affinities [30] at the tβ6-m23 receptor (isoprenaline log K_i_ −5.03 ± 0.17, propranolol log K_i_ −7.62 ± 0.15, CGP 20712A log K_i_ −6.19 ± 0.14; n = 5 for each ligand in this study). The addition of 200 nM mAb3 resulted in a reduction in the specific binding of [^3^H]-CGP 12177 (54.4 ± 1.1% of the specific binding in the absence of mAb3; n = 5). The addition of mAb3 did not significantly change the affinity of the antagonists propranolol or CGP 20712A (log K_i_ values −7.96 ± 0.11, −6.64 ± 0.15, respectively; n = 5, p > 0.05 unpaired *t*-test). However, mAb3 appeared to produce a small but significant increase in the affinity of isoprenaline (log K_i_ with 200 nM mAb3 −5.63 ± 0.10; n = 5, p < 0.05 compared to isoprenaline K_i_ in absence of mAb3, unpaired *t*-test).

Fitting the isoprenaline curves in Fig. 5b with different or shared IC_50_ or B_MAX_ values also confirmed a significant decrease in B_MAX_ (p < 0.0001) and a small increase in affinity for isoprenaline in the presence of mAb3 (log IC_50_ values of −4.3 and −4.9 in the absence and presence of 200 nM mAb3; p < 0.0001; Partial F-test, Prism 6). For the propranolol data, a similar analysis of Fig. 5b only produced a significant reduction in B_MAX_ in the presence of 200 nM mAb3 (p < 0.0001; Partial F-test). However, analysis of the CGP20712A curves indicated a small decrease in IC_50_ (log IC_50_ of −5.9 in the presence of 200 nM mAb3 compared to −5.5 in its absence; p < 0.05) and a marked reduction in B_MAX_ (p < 0.001; Partial F-test).

### 3.4 Measuring mAb3 binding with NanoBRET in HEK293 cells

**Fig. 6:**
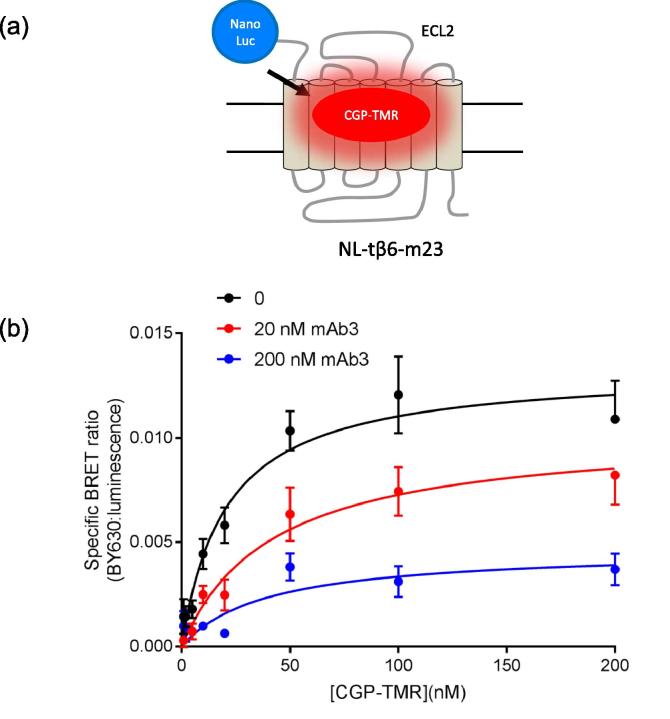
(a) Schematic showing CGP 12177-TMR (CGP-TMR) binding to (NanoLuc tagged NL-tβ6-m23 using NanoBRET. (b) Saturation binding of increasing concentrations of CGP-12177-TMR (CGP-TMR) in the absence and presence of 20 nM or 200 nM mAb3. Non-specific binding was defined in the presence of 10 µM propranolol. Data points represent mean ± s.e.m. from four separate experiments where each experiment was performed in duplicate.

Proximity-based assays have provided a platform to monitor GPCR function and dimerization with great success [40], [41]. Recent advances in the brightness and stability of luciferases (e.g. NanoLuc, [42]) have facilitated the use of bioluminescence resonance energy transfer (BRET) to monitor fluorescent ligand binding at GPCRs [29], [43], and at a receptor tyrosine kinase [44]. Here we have utilised an N-terminal NanoLuc-tagged tβ6-m23 receptor (NL-tβ6-m23) expressed in HEK 293 cells to measure the binding of the fluorescent ligand CGP-12177-TMR [29], [43], [45] to the turkey β_1_-adrenoceptor expressed in HEK 293 cells (Fig. 6a). CGP-12177-TMR showed clear specific binding to the NL-tβ6-m23 receptor (Fig. 6b). These data provided an estimate for the K_D_ value of CGP-12177-TMR of 17.8 ± 4.0 nM (n = 4, Fig. 6b). Incubation of CGP-12177-TMR with 20 nM mAb3 resulted in no significant effect on the affinity of CGP-12177-TMR (K_D_ 31.1 ± 11.2; n = 4, paired *t*-test) or on the specific BRET ratio (87.7 ± 10.9% B_max_; n = 4, paired *t*-test, Fig. 6b) The addition of mAb3 at *circa* ten times K_D_ (200 nM) resulted in a significant decrease in the specific BRET ratio (39.9 ± 9.3% B_max_; n = 4, p < 0.05 paired *t*-test, Fig. 6b) without any significant effect on the affinity of CGP-12177-TMR (K_D_ with 200 nM mAb3 39.3 ± 11.4 nM; n = 4, p > 0.05 paired *t*-test).

**Fig. 7:**
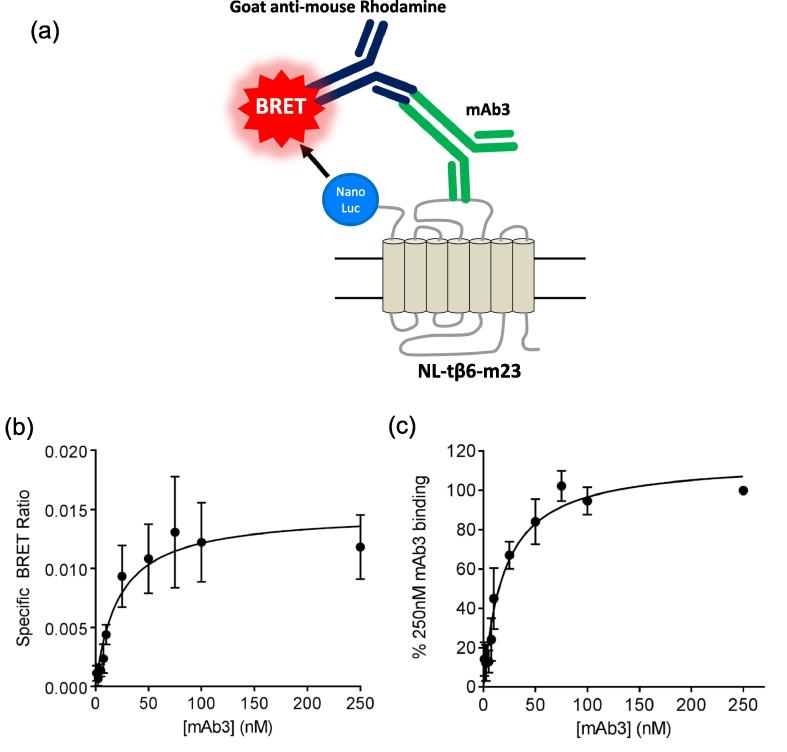
(a) Schematic showing MAb3 binding to the NL-tβ6-m23 detected using a fluorescently-labelled secondary goat anti-mouse antibody that recognises the murine mAb3. BRET occurs between the Nanoluc and the rhodamine fluorophore of the secondary antibody. (b, c) NanoBRET signal obtained from fixed HEK 293 cells (which had been previously transfected with the NL-tβ6-m23 receptor) following incubation with increasing concentrations of mAb3 and subsequent exposure to a rhodamine-labelled goat anti-mouse secondary antibody. Non-specific binding was determined in the presence of the secondary antibody alone. Data points are mean ± s.e.m. of four separate experiments where each experiment was performed in duplicate. Data are expressed (b) as the mean specific BRET ratio (after subtraction of the secondary antibody control values) obtained in the four experiments at each concentration of mAb3 or (c) as a % of the binding obtained with 250 nM mAb3 in each individual experiment.

NanoBRET was also used to quantify the direct binding of mAb3 to the NL-tβ6-m23 receptor. In these experiments, mAb3 was allowed to bind the turkey ECL2, and was then treated with a fluorescently-conjugated secondary antibody. Upon luciferase substrate addition, the BRET between the luciferase and a rhodamine-tagged secondary antibody (bound to mAb3) was then quantified (Fig. 7a). The concentration of the secondary rhodamine-tagged antibody was kept constant across all conditions. Increasing concentrations of mAb3 resulted in an increase in the BRET ratio with a clear saturable component (Fig. 7b, c). From these data, the affinity of mAb3 for the NL-tβ6-m23 receptor in HEK cells was calculated to be 19.5 ± 4.7 nM (n = 4, Fig. 7c).

### 3.5 Effect of mAb3 on β_1_-adrenoceptor-mediated G_s_ signalling in stable cell lines

The tβ_1_-AR couples to G_s_ proteins, and therefore activation of the tβ_1_-AR results in an increase in intracellular cAMP and activation of the protein kinase A signalling pathway [31], [37]. Here, we examined the potential for mAb3 to stimulate CRE-SPAP gene expression or [^3^H]-cAMP accumulation in the presence of 1 mM of the phosphodiesterase inhibitor IBMX via the tβtrunc and tβ6-m23 receptors when stably expressed in CHO cells.

**Fig. 8:**
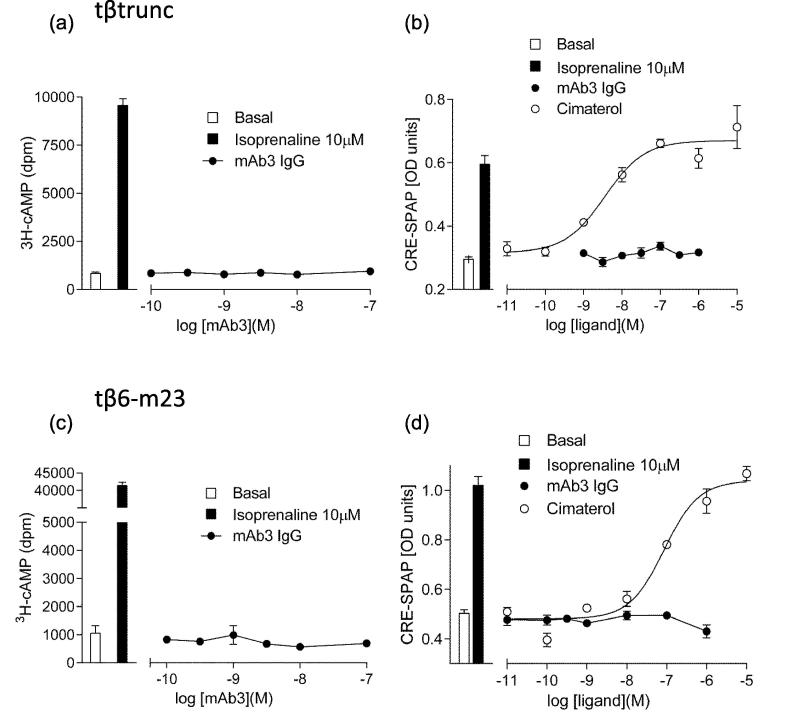
[^3^H]-cAMP accumulation and CRE-mediated SPAP production in CHO cells expressing the (a-b) tβtrunc or (c-d) tβ6-m23 receptor. (a,c) [^3^H]-cAMP accumulation in response to mAb3. (b,d) CRE-mediated SPAP production in response to mAb3 and cimaterol. Bars indicate basal (a,c) [^3^H]-cAMP accumulation, (b,d) basal CRE-mediated SPAP production, and that in response to 10 μM isoprenaline. Data are mean ± s.e.m. of triplicate determinations in a single experiment. These single experiments are representative of (a,c,d) five, or (b) eleven separate experiments.

The full agonist isoprenaline (10 μM) was able to stimulate an increase of [^3^H]-cAMP over basal levels after a five hour incubation (tβtrunc 6.25 ± 0.35-fold, n = 5; tβ6-m23 27.50 ± 4.28-fold, n = 21; Fig. 8a, c). Similarly, 10 μM isoprenaline resulted in an increase in CRE-mediated SPAP production of 1.92 ± 0.07 (n = 33) and 2.04 ± 0.05 (n = 20) fold over basal for tβtrunc and tβ6-m23 receptors, respectively (Fig. 8b, d). MAb3 was unable to stimulate a change in [^3^H]-cAMP or CRE-mediated SPAP production at either the tβtrunc or tβ6-m23 receptors (Fig. 8, p > 0.05 comparing response of mAb3 to basal [^3^H]-cAMP accumulation or SPAP production).

### 3.6 The effect of mAb3 on agonist-induced signalling in stable cell lines expressing the at the turkey β_1_-adrenoceptor

**Fig. 9:**
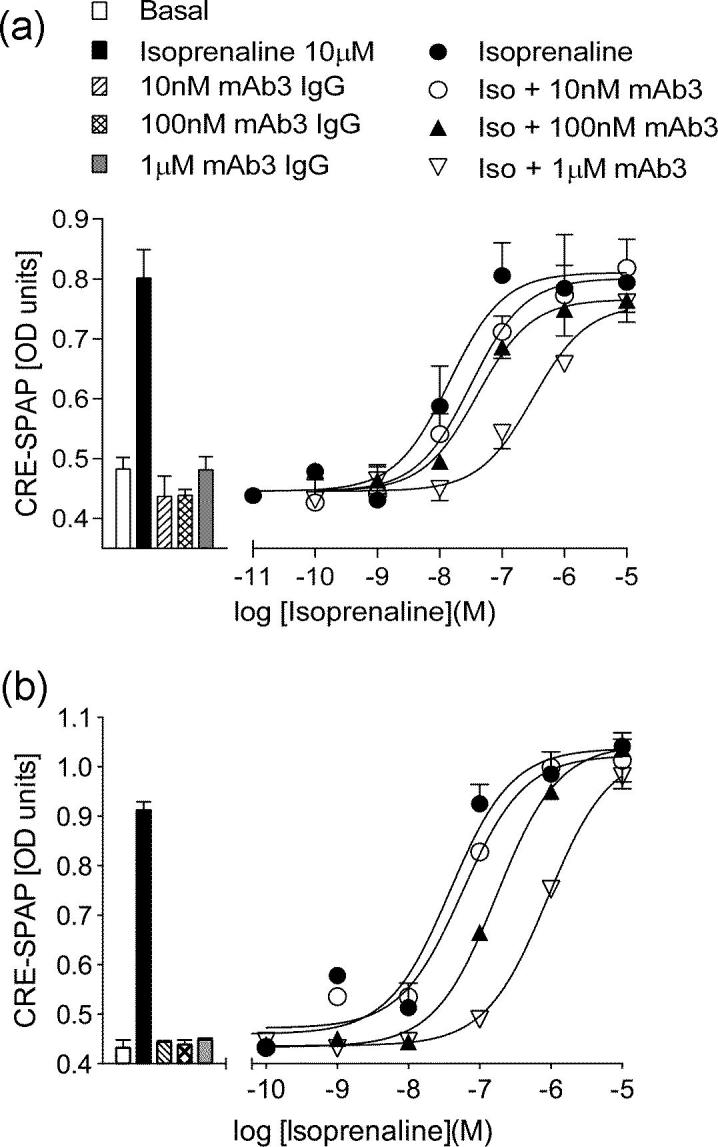
CRE-mediated SPAP production in CHO cells expressing the (a) tβtrunc or (b) tβ6-m23 receptor in response to isoprenaline in the absence and presence of 10 nM, 100 nM and 1 μM mAb3. Bars represent the basal SPAP production, and that in response to 10 nM, 100 nM and 1 μM mAb3. Data points are mean ± s.e.m. of triplicate determinations in a single experiment and are representative of five separate experiments.

To investigate whether mAb3 could antagonise isoprenaline–stimulated responses, CHO cells stably expressing the tβtrunc or tβ6-m23 receptors were stimulated with a range of concentrations of isoprenaline in the absence and presence of mAb3. Thirty minute pre-incubation with mAb3 was able to antagonise isoprenaline-mediated SPAP production at both the tβtrunc (Fig. 9a) and tβ6-m23 (Fig. 9b) receptors in a concentration-dependent manner, resulting in a series of rightward shifts in the isoprenaline concentration-response curves and a reduction in E_max_ (Table 3). Schild analysis gave Hill slopes of 0.91 ± 0.14 at the tβtrunc, and 0.68 ± 0.05 at the tβ6-m23 receptors. The value obtained at the tβ6-m23 receptor significantly differed from unity (p < 0.05, unpaired *t*-test comparing to unity), which suggested a non-competitive interaction between mAb3 and isoprenaline. It is clear from the radioligand binding studies (above) that mAb3 is acting allosterically on a sub-population/conformation of the β_1_AR. It is therefore likely that the antagonism of functional responses to isoprenaline by mAb3 is due to the gradual loss of this population of β_1_AR. For these reasons, functional data were also analysed using equations for an allosteric modulator derived from the functional model previously described [36], [46]. First, the relative activities of the isoprenaline concentration response curves in the presence and absence of mAb3 were calculated. Regression analysis of these relative activity values was then used to obtain an estimate for the affinity of mAb3. The resulting affinity of mAb3 was similar at the two turkey receptors (tβtrunc log K_B_ −7.39 ± 0.11, n = 5; tβ6-m23 log K_B_ −7.50 ± 0.09, n = 5), which share good agreement with the affinity of mAb3 at these receptors measured with radioligand binding.

**Table 3:** Log EC_50_ values, concentration ratios, relative activity, and E_max_ as a percentage of 10 μM isoprenaline-stimulated CRE-SPAP production in CHO tβtrunc or tβ6-m23 CRE-SPAP cells. Cells were treated with isoprenaline in combination with differing concentrations of mAb3 for five hours. Concentration ratios were determined by dividing the EC_50_ in the presence of mAb3 from that in its absence. Relative activity represents the effect mAb3 has on the potency and efficacy of isoprenaline, and was derived as described under Methods. Data for each condition are mean ± s.e.m. from five individual experiments performed in triplicate. ^*^p < 0.05 comparing E_max_ to 100%, or relative activity to a value of 1, unpaired *t*-test.

Ligand	tβtrunc				tβ6-m23			
	Log EC_50_	Concentration Ratio	E_max_ % max	Relative activity	Log EC_50_	Concentration Ratio	E_max_ % max	Relative activity
Isoprenaline	−8.32 ± 0.15	–	100	1	−7.24 ± 0.13	–	100	1
Iso + 10 nM mAb3	−8.30 ± 0.24	1.31 ± 0.28	109 ± 8	0.87 ± 0.11	−7.11 ± 0.13	1.50 ± 0.27	96 ± 5	0.77 ± 0.12^*^
Iso + 100 nM mAb3	−7.88 ± 0.16	2.75 ± 0.32	96 ± 8	0.30 ± 0.05^*^	−6.69 ± 0.14	3.84 ± 0.34	90 ± 6	0.26 ± 0.03^*^
Iso + 1 μM mAb3	−7.04 ± 0.15	21.99 ± 6.2	82 ± 10	0.05 ± 0.01^*^	−5.92 ± 0.14	24.39 ± 4.1	70 ± 9^*^	0.04 ± 0.01^*^

**Fig. 10:**
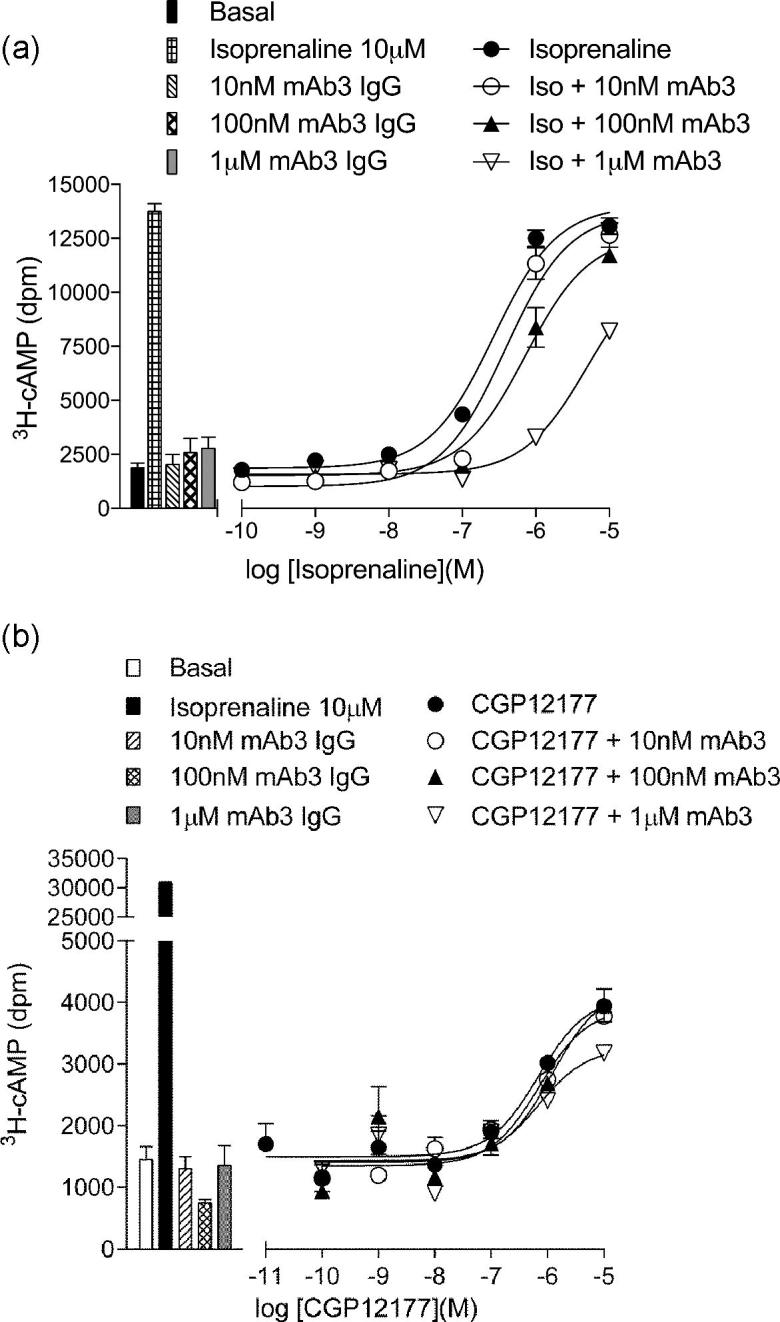
[^3^H]-cAMP accumulation in CHO tβ6-m23 cells in response to (a) isoprenaline or (b) CGP 12177 in the absence and presence of 10 nM, 100 nM and 1 μM mAb3 for five hours. Bars represent the basal [^3^H]-cAMP accumulation, and that in response to 10 nM, 100 nM and 1 μM mAb3. Data points are mean ± s.e.m. of triplicate determinations in a single experiment and are representative of five separate experiments.

The same effect was observed when measuring [^3^H]-cAMP accumulation in the high expressing tβ6-m23 cell line in the presence of 1 mM IBMX. Increasing concentrations of mAb3 resulted in rightward shifts and reduction in E_max_ in the concentration-response curves of isoprenaline after a five hour incubation (mAb3 log K_B_ −7.75 ± 0.12, n = 5; Fig. 10a, Table 4). Interestingly, mAb3 did not have any effect on the partial agonist response of CGP 12177 (Fig. 10b; Table 4).

**Table 4:** Log EC_50_ values, concentration ratios, relative activity and E_max_ as a percentage of 10 μM isoprenaline-stimulated [^3^H]-cAMP accumulation in CHO tβ6-m23 CRE-SPAP cells in response to five hour stimulation with isoprenaline or CGP 12177 in combination with differing concentrations of mAb3. Concentration ratios were determined by dividing the EC_50_ (in M) in the presence of mAb3 from that in its absence. Data for each condition are mean ± s.e.m. from five individual experiments performed in triplicate. ^*^p < 0.05 comparing E_max_ to 100%, or relative activity to a value of 1, unpaired *t*-test.

Ligand	Isoprenaline				CGP 12177		
	Log EC_50_	Concentration Ratio	E_max_ % iso max	Relative activity	Log EC_50_	Concentration Ratio	E_max_ % iso max
Ligand	−6.61 ± 0.13	–	100	1	−6.03 ± 0.21	–	9 ± 3
Ligand + 10 nM mAb3	−6.51 ± 0.12	1.53 ± 0.22	106 ± 4	0.69 ± 0.11^*^	−6.22 ± 0.29	0.81 ± 0.40	5 ± 3
Ligand + 100 nM mAb3	−5.98 ± 0.10	5.24 ± 0.60	86 ± 6^*^	0.19 ± 0.04^*^	−6.12 ± 0.14	0.74 ± 0.36	5 ± 3
Ligand + 1 μM mAb3	−5.45 ± 0.12	17.68 ± 2.35	57 ± 4^*^	0.03 ± 0.01^*^	−6.23 ± 0.04	0.83 ± 0.21	4 ± 2

### 3.7 MAb3 induces the dissociation of isoprenaline from the receptor

**Fig. 11:**
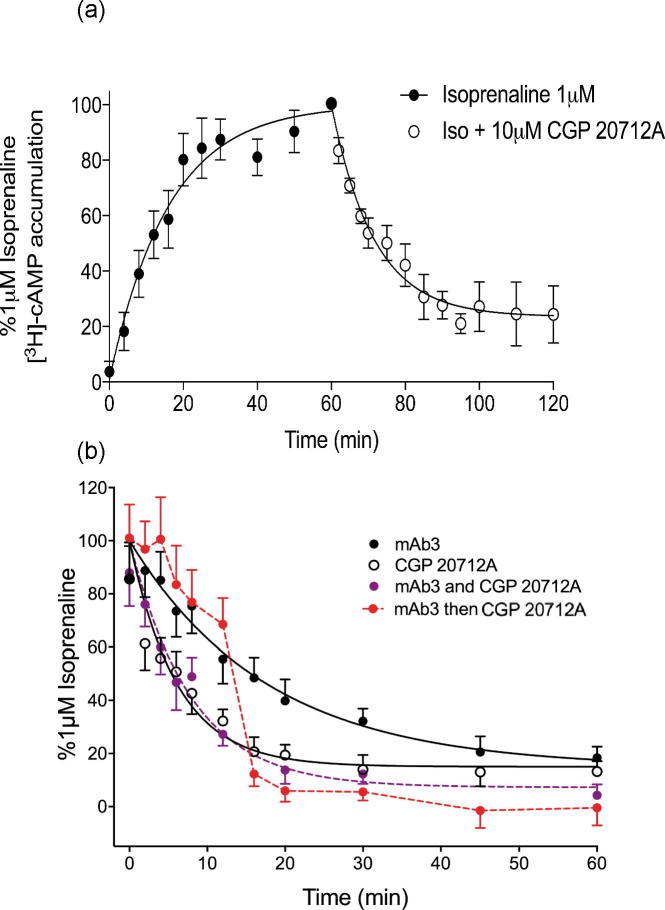
(a) Intracellular [^3^H]-cAMP accumulation in CHO tβ6-m23 cells in response to 1 μM isoprenaline for the first 60 min followed by 1 μM isoprenaline with 10 μM CGP 20712A. (b) Intracellular [^3^H]-cAMP accumulation in CHO tβ6-m23 cells in response in response to 1 μM isoprenaline for the first 60 min followed by 1 μM mAb3, 10 μM CGP 20712A, 1 μM mAb3 plus 10 μM CGP 20712A, or 1 μM mAb3 for the first twelve minutes of the washout period, followed by 1 μM mAb3 in combination with 10 μM CGP 20712A. Ligands were added after one hour stimulation with 1 μM isoprenaline. Data are expressed as a percentage of intracellular [^3^H]-cAMP stimulated by 1 μM isoprenaline for 60 min. Data points are combined mean ± s.e.m. of from five separate experiments, each performed in duplicate.

To investigate whether mAb3 binding to ECL2 was truly acting as (a) a negative allosteric regulator or (b) sterically interfering with the association or dissociation of ligands from the orthosteric binding site of the thermo-stabilised β_1_AR-m23 StaR, we investigated the effect of mAb3 on the kinetics of the intracellular cAMP response to isoprenaline. Isoprenaline (1μM) caused an increase in the intracellular [^3^H]-cAMP concentration until equilibrium was reached after *circa* thirty minutes (Fig. 11a). The addition of 10 μM CGP 20712A at equilibrium resulted in a sharp decrease in the concentration of intracellular [^3^H]-cAMP that could be fitted to a one-phase exponential decay curve (Fig. 11a). This was quantified by calculating an apparent rate constant for this effect (0.118 ± 0.012 min^−1^; n = 5) that reflects the rate for the dissociation of isoprenaline from the ligand binding pocket and the subsequent breakdown of [^3^H]-cAMP. In contrast, the addition of serum-free media without any additional ligand did not result in any significant change in the intracellular levels of [^3^H]-cAMP suggesting that there was no significant dissociation of isoprenaline from the receptor over this time period (p > 0.05, unpaired *t*-test comparing 1 μM isoprenaline with serum free media to that of 100% obtained in the presence of 1 μM isoprenaline alone; n = 5; data not shown).

1 μM mAb3 was able to cause a similar decrease in intracellular [^3^H]-cAMP with a rate constant of 0.046 ± 0.001 min^−1^ (n = 5; Fig. 11b). This was significantly different from the rate constant obtained with CGP 20712A (p < 0.05, unpaired *t*-test). In combination, 1 μM mAb3 and 10 μM CGP 20712A resulted in a high rate constant (0.108 ± 0.011 min^−1^, n = 5) that was not significantly different from that obtained with 10 μM CGP 20712A alone (p > 0.05, unpaired *t*-test; Fig. 11b). Cells were also treated with 1 μM mAb3 for the first sixteen minutes prior to the addition of 10 μM CGP 20712A. As can be seen in Fig. 11b, the initial reduction in intracellular [^3^H]-cAMP with mAb3 followed a similar time course to that previously seen with 1 μM mAb3. However, upon subsequent addition of 10 μM CGP 20712A in combination with 1 μM mAb3 (t = 16 onwards) the profile of the fall in intracellular [^3^H]-cAMP accelerated to match that of 10 μM CGP 20712A alone.

## 4 Discussion

MAb3 was originally generated against a thermo-stabilised β_1_AR-m23 StaR [28]. Here, we have demonstrated specific binding of mAb3 to the purified turkey β_1_AR-m23 StaR protein. Furthermore, binding of mAb3 to β_1_AR-m23 StaR protein was significantly inhibited by a turkey β_1_-AR ECL2 peptide confirming that the likely epitope for the antibody was in ECL2 as originally proposed by Hutchings et al. [28]. However, it was notable that the human ECL2 peptide did not attenuate mAb3 binding.

Two CHO cell lines expressing different mutant turkey β_1_-adrenoceptors were used to investigate the pharmacology of mAb3 in intact cellular systems. These were cells stably expressing either a C-terminal truncated wild-type turkey β-adrenoceptor (tβtrunc; [31]) or a variant containing m23 thermo-stabilising mutations (tβ6-m23; [30]). The binding of mAb3 resulted in a concentration-dependent attenuation of the specific binding of [^3^H]-CGP 12177 to both tβ_1_-ARs. Applying an allosteric ternary complex model to these data enabled an estimate of the affinity of mAb3 for both the low expressing tβtrunc and high expressing tβ6-m23 receptors to be determined. This did not significantly differ between the two tβ_1_-AR variants (the dissociation constant K_B_ was *circa* 20–25 nM in both cell lines). The primary sequences of the ECL2 in these two receptors are identical, implying that m23 mutations present in the tβ6-m23 receptor did not significantly change the conformation of the ECL2. The cooperativity factor between mAb3 and [^3^H]-CGP 12177 was also similar for the two tβ_1_-ARs and indicated a high degree of negative cooperativity in both cases (Table 2). These data are consistent with a previous suggestion that mAb3 may behave as an allosteric modulator of [^3^H]-dihydroalprenolol binding [28].

The specific binding of mAb3 to CHO cells expressing β_1_-ARs was also visualised using immunocytochemistry. These data confirmed that mAb3 could selectively detect only tβ_1_-adrenoceptors (both tβtrunc and tβ6-m23 variants) in intact cells and was not able to bind to the human β_1_-adrenoceptor. It was also able to detect tβtrunc at very low expression levels (92 ± 14 fmol/mg protein) and mAb3 binding could be inhibited by a turkey ECL2 peptide, but not by the equivalent human ECL2 peptide.

The human ECL2 shares 70% homology with the Tk ECL2 (Fig. 4d). The human β_1_-AR ECL2 primary sequence differs from the Tk ECL2 by only eight residues. Single point mutations of these turkey residues to their human equivalent did not affect the binding of mAb3 in cells transiently transfected with the relevant variant of the t β6-m23 receptor. All showed a 38–60% reduction on the specific binding of 0.5–2.5 nM [^3^H]-CGP 12177 in the presence of 1 μM mAb3. However, a triple mutant (D186S, P187D, Q188E) abolished the negative effect of mAb3 on the binding of [^3^H]-CGP12177 to the t β6-m23 receptor. Interestingly, substitution of the full human ECL2 sequence in place of the turkey ECL2 in tβ6-m23 showed the same effect as the triple mutant, suggesting that residues D186, P187 and Q188 are necessary for mAb3 binding. Mapping these residues to the β_1_AR-m23 StaR crystal structure (PDB ID: 2Y03; [39]) indicate that the majority of these residues are located on the extracellular facing surface of the ECL2 (Fig. 4). It is worth pointing, however, that mutation the three residues to their human equivalents (D186S, P187D, Q188E) may also alter the three dimensional packing of ECL2, and that this could mask the true mAb3 binding epitope. However, either way, these mutations do explain the species selectivity of mAb3.

The potential allosteric nature of the interaction of mAb3 with the turkey β_1_-AR was further investigated using ligand-binding assays. CHO tβ6-m23 cells were incubated with increasing concentrations of [^3^H]-CGP 12177 in the absence and presence of mAb3. In these experiments the K_D_ of [^3^H]-CGP 12177 was not significantly changed by the presence of 200 nM mAb3. However, the addition of 200 nM mAb3 did produce a significant 42% reduction in the maximal specific binding capacity of [^3^H]-CGP 12177. This suggests that mAb3 had effectively ‘removed’ a significant proportion of the β_1_-adrenoceptor population that is available to bind [^3^H]-CGP 12177. Competition binding assays using 0.4–1.8 nM [^3^H]-CGP 12177 and a range of orthosteric ligands (in the presence or absence of mAb3) were consistent with this conclusion. Thus, the affinities of CGP 12177 and CGP 20712A obtained in the presence of 200 nM mAb3 were similar to those obtained in the absence of mAb3, with a small increase in the affinity of isoprenaline. There was, however, a significant decrease (46%) in the specific binding capacity of [^3^H]-CGP 12177 in the absence of the orthosteric ligands.

To gain some insight into the percentage of receptors occupied by mAb3 at the concentrations used in the present study, we have used a NanoBRET binding assay [43]. Using a similar approach to that we have previously reported in HEK 293 cells to study human β_1_- and β_2_-adrenoceptors [29], [43], we demonstrated that a fluorescent analogue of CGP 12177 (CGP-12177-TMR) could be used to monitor the effect of mAb3 on the K_D_ and maximal specific binding capacity of CGP-12177-TMR to an N-terminal NanoLuc-tagged variant of the tβ6-m23 receptor. These data showed a similar 40% reduction in maximal specific binding in the presence of 200 nM mAb3 without any significant effect on the affinity of CGP-12177-TMR. NanoBRET was also used to monitor directly the affinity of mAb3 for NL-tβ6-m23 expressed in HEK 293 cells using NanoBRET between a rhodamine-labelled secondary antibody to mAb3 and the extracellular Nanoluc of NL-tβ6-m23. The affinity of mAb3 measured at the NL-tβ6-m23 receptor (K_D_ 17.8 nM) was very similar to that (19.5 nM) estimated from radioligand binding studies at the tβ6-m23. The large size of the mAb3-secondary antibody complex does, however, mean that we can only conclude that the rhodamine on the secondary antibody was in close proximity (<10 nm) to the NanoLuc of a neighbouring β_1_-adrenoceptor rather than definitively conclude that it is to the β_1_-adrenoceptor to which mAb3 was bound. Notwithstanding these uncertainties, we can conclude that the occupancy achieved at the turkey β_1_-adrenoceptor with 200 nM and 1 µM mAb3 was very high (92 and 98% respectively).

Taken together, these data suggest that mAb3 binds to ECL2 of the turkey β_1_-adrenoceptor and acts allosterically to prevent the binding of orthosteric ligands to a subset (*circa* 40%) of β_1_-receptors present in the cells. The interaction of mAb3 with ECL2 and its consequent allosteric effects are consistent with the important role that ECL2 appears to have in ligand recognition [17], [18], [19] and as a site of allosteric modulation for other GPCRs [20], [21]. Furthermore, the structure and position of the ECL2 is known to be critical for receptor activation [22], [23].

To investigate the functional impact of mAb3 in stable CHO cell lines expressing the tβtrunc or tβ6-m23 variants of the turkey β_1_-adrenoceptor we also investigated their signalling characteristics. We found no evidence that mAb3 was able to stimulate cAMP accumulation or downstream cAMP response element reporter gene responses. This contrasts with previous data on mAb3 in transiently transfected cells, where a small cAMP response was reported [28]. This is most likely due to the very high expression levels achieved with transient transfection. It has been documented previously that high receptor density can reveal partial agonist behaviour of ligands previously characterised as antagonists [47], [48]. MAb3 was, however, able to antagonise isoprenaline-mediated activation of the two tβ_1_-ARs. This was evident both at the level of cAMP accumulation and a downstream reporter gene transcription. Schild analysis was consistent with a non-competitive interaction between mAb3 and isoprenaline at the tβ6-m23 receptor. The reduction in the number of available receptors in the presence of mAb3 (detected from binding studies with both [^3^H]-CGP 12177 and the fluorescent analogue of CGP 12177) that can bind agonist almost certainly explains the effect on signalling. Thus, the shift of the concentration-response curve to isoprenaline to higher agonist concentrations produced by mAb3 can be explained by: (a) the reduced receptor number and (b) the ability of the receptor reserve (due to signalling amplification) to enable maximal functional responses to isoprenaline to be achieved following a reduction in the receptor population. The data obtained with mAb3 are consistent with an allosteric effect. However, it is also possible that the interaction of a bulky monoclonal antibody with ECL2 might restrict access of agonists and antagonists to (or from) the orthosteric binding site deep within the transmembrane regions of the β_1_-adrenoceptor [8], [39], due to a steric hindrance effect.

To investigate this possibility, the temporal profile of the changes in intracellular cAMP levels was monitored following sequential addition of isoprenaline and mAb3 or CGP 20712A. Addition of CGP 20712A, after intracellular cAMP levels had reached an equilibrium level following initial addition of isoprenaline, led to a rapid fall in cellular cAMP levels. This was due to the continuous binding and dissociation of isoprenaline that occurs when equilibrium is reached, and the rapid occupancy of vacant orthosteric binding sites that occurs when a high concentration of the competitive antagonist CGP 20712A is subsequently added. Addition of mAb3 led to a slower fall in cAMP levels that is consistent with a negative allosteric interaction. In the absence of mAb3 or CGP 20712A, cAMP levels were maintained for 60 min. Simultaneous addition of mAb3 and CGP 20712A provided the same rapid fall in cAMP to that obtained with CGP 20712A alone. These data indicate that mAb3 did not reduce access of CGP 20712A to, or isoprenaline from, the orthosteric binding site. This was confirmed when mAb3 was added first, to establish a slow fall in intracellular cAMP, and then subsequent addition of CGP 20712A created a rapid fall in cAMP levels.

It is very clear is that at the highest concentration of mAb3 used, the allosteric effect of the antibody on [^3^H]-CGP 12177 binding is confined to a subset/subpopulation of β_1_-ARs. The lack of effect of this high concentration of mAb3 on the ligand affinity (isoprenaline, propranolol, CGP 20712A) for the remaining [^3^H]-CGP 12177 specific binding sites is consistent with mAb3 only interfering with a specific conformation/sub-population of the β1-ARs. The effect of mAb3 on β_1_-AR signalling suggests that the conformation/sub-population of β_1_-AR targeted by the antibody is involved in Gs-mediated agonist action. However, it remains to be established whether this conformation (sensitive to inhibition by mAb3) represents the Gs-coupled β_1_-AR conformation.

In summary, the data obtained in the present study suggest that mAb3 interacts with ELC2 to produce a pronounced negative allosteric effect on the binding of orthosteric ligands to a subset of turkey β_1_-adrenoceptors. It also elicited a negative allosteric effect on functional Gs-mediated responses. The identity of the subset of turkey β_1_-adrenoceptors influenced by mAb3 remains to be established. It is well known that GPCRs can exist in a spectrum of conformations, ranging from resting R and active R∗ states to oligomeric complexes involving G protein, β-arrestin, scaffolding proteins [49], [50] and multiple GPCR molecules [51]. These conformations have the potential to be selectively targeted by antibodies. For example, single-domain camelid immunoglobulins (termed nanobodies) have been utilised to recognise and stabilise active (Nb80; [9], [52], [53]) and inactive (Nb60; [53]) conformations of the β_2_-adrenoceptor.

## Conflict of interest

AB, GC and CJH are shareholders in Heptares Therapeutics (part of the Sosei Group) and hold stock options in the Sosei Group. CJH is a consultant for Heptares Therapeutics. The remaining authors declare no conflict of interest.

## Author contributions

Participated in research design: Soave, Brown, Hutchings, Woolard, Hill.

Conducted experiments: Soave, Cseke.

Performed data analysis: Soave, Hill.

Wrote or contributed to the writing of the manuscript: Soave, Brown, Hutchings, Woolard, Hill.
